# Using qualitative workflow mapping to plan for implementation of pediatric vaccine promotion intervention and trial

**DOI:** 10.1016/j.conctc.2026.101701

**Published:** 2026-10-17

**Authors:** Princilla A. Minkah, Jillian Bongiorni, Melissa Goulding, Milagros C. Rosal, Stephenie C. Lemon, Grace W. Ryan

**Affiliations:** a Division of Preventive and Behavioral Medicine, Department of Population and Quantitative Health Sciences, University of Massachusetts Chan Medical School, 55 Lave Avenue North, Worcester, MA, 01655, USA; b Baystate General Pediatrics, 140 High Street, Springfield, MA, 01105, USA

**Keywords:** Workflow mapping, Formative research, Pediatrics, Vaccination, Qualitative research

## Abstract

To inform a pediatric clinic-based vaccine promotion trial, we conducted workflow mapping interviews with 8 providers and staff at two pediatric clinics. Using an interview guide, participants described the vaccine delivery process and shared perspectives on barriers and facilitators. Interviews were recorded, transcribed, and analyzed using rapid qualitative methods. We created visual workflow maps that guided intervention refinement and tailored implementation protocols for our pilot cluster RCT. We recommend integrating workflow mapping into intervention planning timelines to ensure contextual fit. Rapid qualitative analysis is an efficient, well-suited method for this type of pre-implementation formative research.

## 1 Introduction

There is strong evidence supporting the effectiveness of clinic-level interventions in promoting vaccine uptake [1], [2], however conducting intervention studies in clinics can be challenging, both in terms of research logistics [3] and implementation itself [4]. Further complicating research and implementation work on vaccine promotion interventions is the fact that the vaccine-landscape is constantly changing with updates to recommendation language as well as public receptivity [5]. In preparation for a pilot randomized control trial of CONFIDENCE, a multicomponent clinic-based intervention to promote pediatric vaccine uptake focused on clinics with a high percentage of patients from underrepresented minority populations, we conducted a qualitative workflow process mapping exercise. Clinical workflow mapping, sometimes referred to as process analysis or process mapping, supports development and implementation of clinic-based interventions [6], [7]. While it is commonly used to describe adaptation processes [8], in quality improvement work, [9], [10], and post-implementation to help describe the process [11], [12], it is less commonly used during the intervention and implementation planning phase. We previously designed CONFIDENCE to promote pediatric COVID-19 vaccine uptake [11], but given the time that had passed between its development, our beta-test, and the beginning of this pilot RCT, we wanted to ensure our intervention and implementation plan still aligned with current clinical practice. Here, we describe results from workflow mapping and how we used these results to adapt both the intervention components and implementation process.

## 2 Methods

We developed CONFIDENCE in Spring 2021 and conducted a beta-test in a single pediatric clinic within a large academic medical center system. Briefly, CONFIDENCE consists of a provider training based in evidence-based strategies to promote vaccination, a poster campaign tailored to the clinic featuring staff and providers sharing personal vaccine narratives, and accompanying parent-facing educational materials [11]. In Table 1 we described these three components in more depth along with our originally intended implementation plans (Insert Table 1 here).Following this successful beta-test, we scaled up the project to conduct a pilot RCT with clinics across Central and Western, MA. the workflow mapping was designed to be the first activity in this pilot RCT with the goal of ensuring alignment of our intended plans with clinic workflows.

**Table 1:** CONFIDENCE intervention components and implementation plan.

Component	Target Audience	Description	Delivered By	Implementation Plan
3-Step Communication Training for Provider	Providers were trained and parents received the communications	Training focused on COVID-19 vaccination on (1) strong presumptive provider recommendation, (2) principles of motivational interviewing, and (3) sharing personal vaccine stories	Research study team delivered the training then providers used the approach with parents	Live training via Zoom. Providers use the 3-step approach with parents during clinic visits; offer training or provide training to MAs
Provider *“My reasons why”* Posters	Parents and patients	Professional portrait of the provider with a personal vaccine story centered on their personal motivations for COVID-19 vaccination	Research study team developed the posters, and clinic staff displayed	Posters displayed in clinics exam rooms featuring providers
Parent Facing Educational Materials	Parents and other caregivers	Trifold fact sheet infographic with Q&A regarding vaccination for 5-17-year old children	Front desk staff, medical assistants, nurses, or providers	Provided in multiple languages (English, Spanish, Portuguese, Vietnamese) and kept them stocked in the exam rooms of the clinic

Between June and September 2023, we conducted semi-structured qualitative interviews using a clinic workflow mapping exercise. The University of Massachusetts Chan Medical School Institutional Review Board approved all study activities. We randomized clinics to either the primary intervention condition or the delayed intervention condition; as per our trial protocol we only conducted workflow interviews in the primary intervention condition clinics. When clinics agreed to participate in CONFIDENCE, they were asked to identify a project lead and a team consisting of providers, nursing staff, medical assistants, and front desk staff. We asked our clinic leaders to support recruitment by providing names and contact information for the project team and to send an introduction email about the project. Our team followed up directly via email or phone to schedule interviews. At time of scheduling, we sent an IRB approved factsheet to participants that included details on their participation. We also verbally confirmed consent to participate and to record the conversation at the time of interview. To be consistent with formative implementation research, our sampling goal was to achieve contextual depth, and we recruited participants purposively to represent the range of staff roles involved in vaccine delivery. We ultimately spoke with four participants from each clinic representing a variety of roles that included patient services representative (i.e., front desk staff), medial assistant, nursing staff, and medical provider.

All interviews were conducted via telephone or web-conferencing, audio-recorded, and transcribed using an AI-auto-transcribe feature. Our goal was to fully understand clinic workflow from the time a patient and family entered through the check-out process. Our interviews began by asking participants to describe this process in as much detail as possible, from their own perspective. We asked them to focus on well-child visits for patients aged 5-17 and used probing questions to encourage them to describe all touchpoints between patients and clinic staff, including front desk staff and medical assistants, as well as the locations in the clinic they spent time. For example, we asked specifically about the ‘path’ patients take through the clinic from the waiting area to the exam rooms and where they stop along the way (e.g., where height and weight measurement takes place). We used notes taken during the interviews, transcripts and audio recordings, to first generate a timeline of the vaccine process and then create a visual workflow map. With our primary focus in creating workflow maps being to adapt and facilitate implementation of CONFIDENCE, our focus was on processes and interactions that occurred within the clinic setting and on the specific individuals and their involvement in the vaccine delivery process. We sent these maps to the clinic teams with the request that they review for accuracy and specificity. Clinic leads provided feedback and we revised maps accordingly (e.g., we had not correctly identified the order in which the resident physician saw patients). We used rapid qualitative analysis (RQA) [12] to analyze the rest of the interview data and explore results in the following domains: (1) potential role of medical assistants and/or front desk staff in intervention, (2) barriers, and (3) current context. RQA is increasingly popular in implementation science research [13], [14] and has high concordance with traditional qualitative analysis methods [15]. RQA involves creating a template to summarize interview data and using this template to summarize data by domain across participants.

## 3 Results

Across two clinics representing two different health systems, one in Central, MA and one in Western, MA, we conducted a total of 8 interviews which lasted on average 16 min. We spoke with providers (n = 5), nursing staff (n = 1), front desk staff (n = 1), and a medical assistant (n = 1). The majority of participants identified as female (n = 7), and the sample was split evenly between the two clinics with four participants from each.

### 3.1 Workflow maps

The process for vaccination in both clinics was similar and therefore we present in Fig. 1 a sample map from one of the clinics. In interviews, participants outlined steps starting from a patient checking in for an appointment through leaving the office. These maps highlight multiple touchpoints with providers and staff that patients have throughout their time in clinic and the numerous individuals involved in the vaccination process (Fig. 1).

**Fig. 1:**
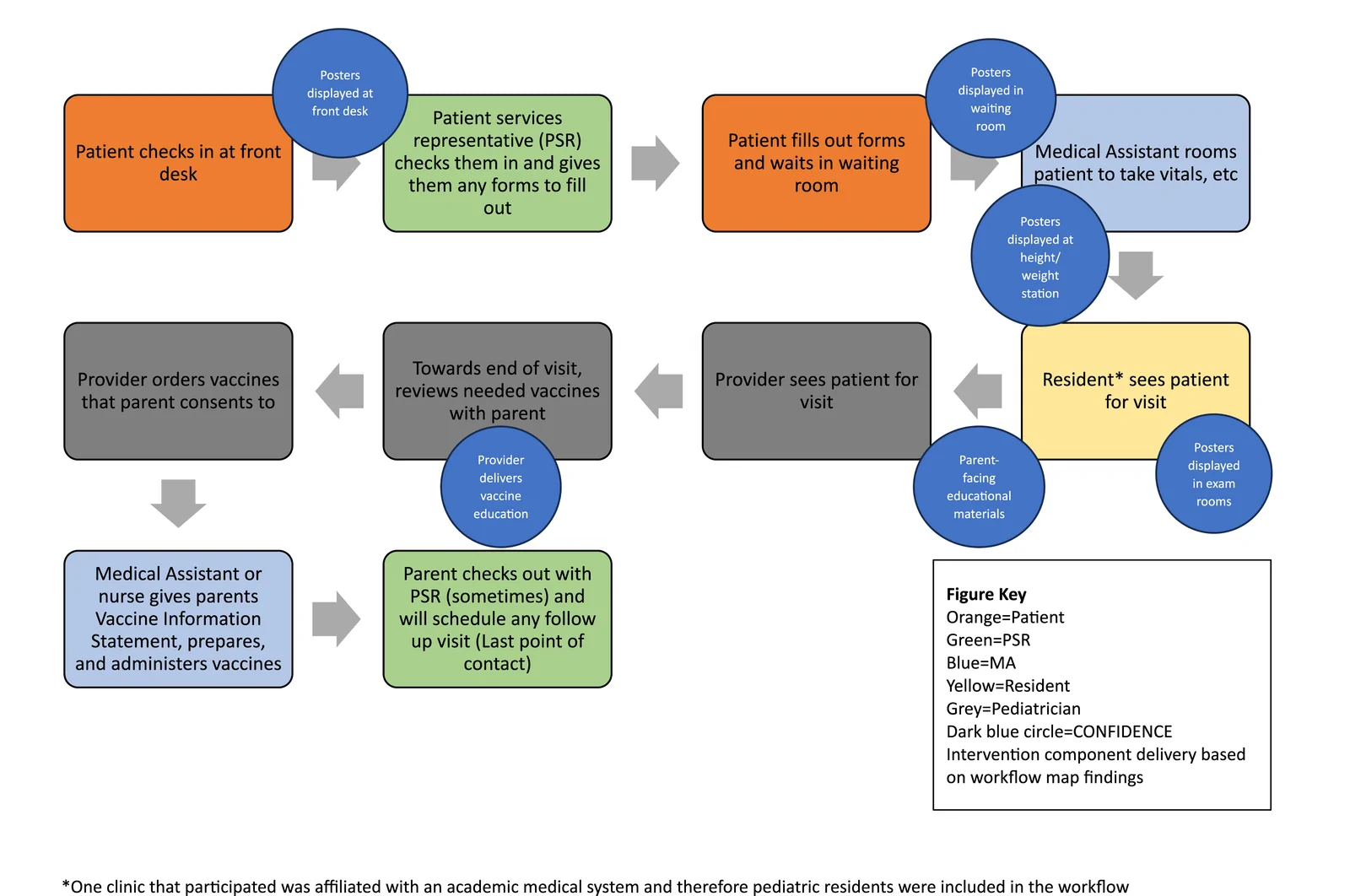
Example workflow map highlighting integration of CONFIDENCE intervention components. *One clinic that participated was affiliated with an academic medical system and therefore pediatric residents were included in the workflow.

### 3.2 Potential role of medical assistants/front desk staff in intervention

Nearly all participants agreed that vaccine promotion was within the scope of both medical assistants and front desk staff's role, however adding any additional tasks to their current roles was not feasible. As one provider reflected “it's not inappropriate for [MAs] we're just so short staffed. I think having them do anything extra is something we're trying to avoid.” Another provider noted that medical assistants and providers are “horribly overworked” but could benefit from training or other education related to vaccine promotion. The primary task that many agreed was feasible was to distribute materials related to the intervention and participate in the poster campaign by providing personal vaccine narratives. Additionally, in describing the project during these interviews, several participants offered that medical assistants may be able to support research-related data collection.

### 3.3 Current issues related to vaccination

Participants noted a variety of current issues related to vaccination in their clinics and addressing vaccine hesitancy that can be split into two sub-themes: (1) issues related to vaccine promotion and (2) logistical challenges for vaccine delivery. While all providers stated that they did not have difficulty with ensuring their patients were up-to-date on school-required vaccines, most reported that the non-mandated vaccines, particularly COVID-19 and influenza, face more resistance. Participants attributed much of this to inaccurate information and noted that it is often heightened among certain cultural or religious groups. For COVID-19 and influenza vaccination many providers reported that parents express concerns about efficacy of the vaccines which is hard to combat and that there “even families where like dad died from COVID or uncle died, they're still hesitant.”

Others reflected on challenges related to accessibility. In both clinics, participants reported that there have been times when they did not have COVID-19 or influenza vaccines in stock and it was challenging and time-consuming to figure out how to connect patients to accessible vaccinating locations (Insert Table 2 here).

**Table 2:** Overview of adaptations to CONFIDENCE intervention and implementation.

Perspectives of participants (n = 8)	Adaptation made to CONFIDENCE implementation or intervention
Medical assistants and front desk staff lack time to participate beyond regular duties	Identified low time-commitment way to facilitate participation by promoting medical assistant and front desk staff participation in poster campaign; original plan of providing training to MAs was determined to be out of scope
Influenza faces as much, if not more, resistance as COVID-19 vaccination	Expand scope of intervention to include and assess both COVID-19 and influenza vaccine hesitancy
Lack of knowledge about alternative vaccination providers	Create locally tailored lists of vaccine locations vetted for accessibility and availability of COVID-19 and influenza vaccines
Medical assistants and front desk staff have multiple touch points with patients and families	MAs and front desk staff can support data collection; front desk staff will hand survey to parent; MA will remind parent about survey; surveys are returned to front desk staff

## 4 Discussion

Using a qualitative workflow mapping exercise facilitated timely and context-relevant adaptations to our intervention and implementation. We completed this exercise as part of our formative research phase for our intervention to promote pediatric COVID-19 vaccination in clinics serving a higher percentage of underrepresented and minority patients and families. Our results identified several key changes including expansion of scope to include influenza vaccination and specific, but limited, roles for medical assistants.

Increasingly workflow mapping is integrated into implementation planning processes and similar to other studies [6], [7], and we found great utility to this. Beyond planning for implementation, we also found it a useful exercise to better facilitate data collection. While this was not an intended goal of the exercise, these findings proved very useful. Our primary outcome for the study is based on parent exit surveys to be filled out at the conclusion of a visit. Given that all clinics have slightly different workflows, we were able to use our results to work with each clinic to best understand how to collect these surveys with minimal workflow disruption.

We initially planned for a larger role for medical assistants and front desk staff however our interviews revealed that this would be difficult. Research on the role of medical assistants in health promotion interventions is mixed, with a recognition that they are a potentially useful group to leverage in this type of work [16], but due to multiple barriers, results are mixed in terms of feasibility [17], [18], [19]. However we were able to identify MAs as having a role in providing content for the posters and as being able to support the research aspects of the project (i.e., data collection of parent exit surveys).

Our findings underscore a need to assess the local context to ensure fit between the planned intervention and needs of the setting. In this case, while all participants agreed that addressing hesitancy related to COVID-19 vaccination was an ongoing need, the majority highlighted that influenza vaccination was also a pressing issue. Given that we had accounted for this process of pre-implementation qualitative research and an adaptation period in our timeline we were able to expand the scope of our intervention; however, we recognize that this may not always be possible. Vaccine hesitancy is a constantly evolving area and thus we felt it was essential to build this into the planning period given that we had designed our original intervention content nearly two years prior. Using rapid qualitative methods supported this process by efficiently generating actionable findings during this formative phase of implementation research, where rapid iteration between data collection and intervention refinement was essential.

This study is not without its limitations. Our results may not reflect current vaccine practices in other clinics. Moreover, it is possible that there are perspectives in each of these clinics that were not captured from potential participants who declined to complete an interview. Our approach did however allow for multiple participants per clinic and we built in a feedback mechanism so participants could reflect on the maps before they were finalized. However, our goal was not to create generalizable results, but to support intervention and implementation planning. Additionally, as per protocol, workflow interviews were conducted only in intervention-arm clinics, so perspectives from control-arm practices are not represented. Finally, we limited our data collection to create the workflow maps to clinic workflow surrounding patient visits, which helped support our goal of identify patient touch points, but it is possible that we may have missed some other key activities that occur that support vaccine delivery (i.e., scheduling patient visits, use of electronic health record portals to share vaccine messaging). Future work could expand this workflow mapping process to focus generally on vaccine promotion rather than explicitly on patient encounters.

## 5 Conclusion

Workflow mapping can help support planning for implementation and clinical trial research but is not routinely done as part of these processes. It may be especially important to integrate workflow mapping into research that involves topic areas that are subject to contextual changes, like vaccination. Our process helped us to identify important adaptations to the content of the intervention itself (i.e., expansion to include influenza vaccination) and process for incorporating medical assistants into the intervention itself as well as a supporting role for data collection. Additionally, we demonstrate that rapid qualitative analysis is a feasible and efficient method for pre-implementation formative work, offering a replicable approach for intervention researchers who face time constraints. When possible, workflow mapping should be planned for early in implementation and clinical trial planning research to ensure alignment with local context and identify and mitigate potential challenges that could emerge during implementation and evaluation.

## CRediT authorship contribution statement

**Princilla A. Minkah:** Writing – review & editing, Writing – original draft, Project administration, Formal analysis, Conceptualization. **Jillian Bongiorni:** Writing – review & editing, Resources, Investigation. **Melissa Goulding:** Writing – review & editing, Validation, Methodology, Conceptualization. **Milagros C. Rosal:** Writing – review & editing, Methodology, Conceptualization. **Stephenie C. Lemon:** Writing – review & editing, Methodology, Funding acquisition, Conceptualization. **Grace W. Ryan:** Writing – review & editing, Writing – original draft, Supervision, Project administration, Methodology, Investigation, Formal analysis, Conceptualization.

## Ethics declaration

Written informed consent to take part in the study and to publish the article has been obtained from all participants or their legal representatives. The privacy rights of participants have been observed.

This study was performed in compliance with relevant laws, regulatory frameworks and guidelines where the research took place. This study was approved by the University of Massachusetts Chan Institutional Review Board. (Approval No. STUDY00000728)

## Funding

This work was supported in part by a research grant from Investigator-Initiated Studies Program of Merck Sharp & Dohme LLC. The opinions expressed in this paper are those of the authors and do not necessarily represent those of Merck Sharp & Dohme LLC. The funder had no role in the design of this study, analysis of data, writing, or the decision to publish this research.

## Declaration of competing interest

The authors declare that they have no known competing financial interests or personal relationships that could have appeared to influence the work reported in this paper.

## Data Availability

The authors do not have permission to share data.
